# Quality of life and patient reported outcomes in the UK Mammo-50 randomised trial of annual versus less frequent mammographic surveillance in people with breast cancer aged 50 years and over

**DOI:** 10.1186/s12955-025-02396-6

**Published:** 2025-07-01

**Authors:** Andrea Marshall, Peter Donnelly, Nada Elbeltagi, Sophie Gasson, Amy Broadfield, Amy Hopkins, Sue Hartup, Lesley Turner, Annie Young, Eila K Watson, Janet A Dunn

**Affiliations:** 1 Warwick Clinical Trials Unit, University of Warwick, Coventry, UK; 2 Torbay and South Devon NHS Foundation Trust, Torquay, UK; 3 St James’s University Hospital, Leeds, UK; 4 Independent Cancer Patients’ Voice, London, UK; 5 Oxford Brookes University, Oxford, UK

**Keywords:** Breast cancer, Mammographic surveillance, Quality of life, Clinical trial

## Abstract

**Background:**

Mammo-50, a randomised phase III trial, demonstrated that for women aged 50 years or older and 3-years post breast cancer diagnosis, less frequent mammograms (2-yearly after conservation surgery; 3-yearly after a mastectomy) were non-inferior to annual mammograms in terms of detection of recurrences, or new breast primaries. It is important to assess Quality of life (QoL) in this population to ensure no detriment is associated with a less frequent mammographic surveillance schedule.

**Methods:**

A mixed methods QoL sub-study was undertaken to explore potential differences between the trial arms in terms of fear of recurrence, QoL and distress levels and to explore patient reported experiences. Participants were asked to complete a questionnaire booklet annually whilst on the trial. Longitudinal random effects regression models were fitted to assess changes in QoL over time and across trial arms. Free text data were collected on participants worries or concerns.

**Results:**

5235 women were randomised between April 2014 and September 2018, from 114 UK sites of which 4488 women (86%) returned a baseline QoL booklet. With a median 5.7 years follow-up (8.7 years post-curative-surgery), no differences between trial arms were identified for any of the QoL scales measured. Themes identified from the free text data included co-morbidities, family problems and side-effects of hormone therapy.

**Conclusions:**

There were no differences in any of the QoL scales between the trial arms of Mammo-50, implying that less frequent mammographic surveillance does not adversely impact participants’ QoL. Women were concerned with co-morbidities or family problems and side-effects of treatment rather than worries about having less frequent mammograms.

**Trial registration:**

ISRCTN48534559, 26 February 2014.

## Background

Breast cancer remains the most common female cancer worldwide with over 55,900 new cases diagnosed each year in the UK [1] and estimated 2,296,840 new cases diagnosed globally in 2022 [2]. In the UK around 82% of women diagnosed with breast cancer are aged 50 years or older; the 5-year survival rate in the UK is around 88% which has improved progressively in the last 30 years [1]. However as women are surviving longer after their diagnosis, follow-up surveillance and quality of life (QoL) becomes increasingly important for patients and the health care system [3].

It is widely known that treatment for breast cancer can cause many physical and emotional effects thus affecting the QoL of patients, and patient reported outcomes measures (PROMs) help to evaluate these [4, 5]. One of the most common emotional effects of breast cancer is fear of recurrence [6]. Evidence shows that long-term breast cancer survivors still experience fear of recurrence even ten years after diagnosis, which has a negative impact on women’s QoL and an increased depression risk [7, 8]. For some women, these concerns have been allayed to some extent by an annual mammogram and routine clinical examination [9, 10].

Mammo-50, a randomised phase III trial, demonstrated that for women aged 50 years or older and 3-years post breast cancer diagnosis, less frequent mammograms (2-yearly after conservation surgery; 3-yearly after a mastectomy) were non-inferior to annual surveillance mammography in terms of clinical outcomes including breast cancer specific survival and recurrence-free interval [11]. These findings indicate that it was safe to de-escalate surveillance mammography for this population of women who were at a lower risk of breast cancer recurrence [11]. However, before reducing mammographic surveillance, it is important to ensure that there is no detriment to QoL associated with a reduced mammographic surveillance schedule.

A mixed methods QoL sub-study was conducted within the Mammo-50 trial to assess whether differences between the two trial arms occurred in terms of fear of recurrence, QoL and distress levels. A secondary aim was to explore additional patient reported worries and concerns.

## Methods

Mammo-50 was a pragmatic multicentre, randomised phase III trial of annual versus less frequent mammography (2-yearly after conservation surgery; 3-yearly after a mastectomy) for women aged 50 years or over at initial diagnosis of invasive or non-invasive breast cancer, and who were recurrence free 3-years post curative surgery (ISRCTN48534559, 26 February 2014) [11]. Full details of the eligibility criteria for the Mammo-50 trial are given elsewhere but all participants gave written informed consent [11]. Patients with bilateral breast cancer including DCIS, known BRCA or genetic mutation, or a previous breast malignancy were excluded from the study. For women with significant mental health problems, or where English was not their first language, it was left to the discretion of the recruiting clinician. Participation in the mixed methods QoL sub-study was optional. Women who consented were asked to complete a QoL booklet at trial entry (baseline) and then annually for seven years (up to ten years post curative surgery). Participants were encouraged where possible to continue to complete the questionnaires after a recurrence, but could withdraw from the QoL sub-study if they felt unable to continue. Demographics, treatment history and clinical outcomes were collected as part of the main trial [11].

### QoL booklet

The QoL booklet was designed in collaboration with patient representatives and the Independent Cancer Patients’ Voice (ICPV) patient and public involvement (PPI) group. It included the Assessment of Survivor Concerns (ASC) scale [12], Warwick Edinburgh Mental Well-being Scale (WEMWBS) [13], the Functional Assessment of Cancer Therapy for breast cancer (FACT-B) [14] and the USA National Comprehensive Cancer Network (NCCN) Distress Thermometer and problem list [15] and also a blank page for free text data.

The ASC scale contains six questions measuring fears about recurrence and health in cancer survivors with responses on a 4 point Likert scale (‘‘not at all’’, ‘‘a little bit’’, ‘‘somewhat’’, or ‘‘very much’’) [12]. The questions are combined into a cancer worry and a health worry subscale.

The WEMWBS is a validated 14 item scale describing patients’ feelings and thoughts with responses on a 5 point scale (1 = None of the time, 2 = Rarely, 3 = Some of the time, 4 = Often, 5 = All the time). A total score is calculated by summing the 14 individual statements. The average population mean score is around 51 but varies according to the population studied [13].

The FACT-B + 4 is a 41 item questionnaire assessing five domains: physical well-being, social/family well-being, emotional well-being, functional well-being and additional concerns. The responses are on a 5-point Likert scale between 0 (Not at all) to 4 (Very much). Domain scores were calculated using the user guide document for this questionnaire with higher scores representing better quality of life [14].

The NCCN Distress Thermometer measures how distressed a patient has been feeling for the past week by circling a number between 0 and 10, 0 meaning no distress and 10 meaning high distress [15]. Scores were categorised into no distress (score = 0), low distress (scores of 1–4), moderate levels of distress (scores of 5–7) and high levels of distress (scores of 8–10). It also includes a problem list where participants identify from the list of physical, practical, family, emotional or spiritual/religious problems any concerns they may be facing and rank the top four in order of ascending difficulty. On advice from the oversight committees, the reasons for any participant reporting high levels of distress (scores of 8-10) were reported back to the clinical team at sites.

In addition, a blank page was included at the end of the QoL booklet where the participants were asked to report any further details about their worries or concerns.

### Sample size

The Mammo-50 trial required 5000 women to be recruited to demonstrate a 3% non-inferiority margin assuming 5 year disease specific survival of 89% with a 2.5% one-sided significance and 90% power. For the QoL sub-study, a minimum sample of 600 participants was needed to allow the detection of a relatively small standardised difference of 0.3 in QoL with 90% power and 5% 2-sided significance level. For example, this would equate to a 7.5% change in QoL assuming a conservative standard deviation of 25%. It was agreed with the trial oversight committees and the PPI group to offer all women entering the Mammo-50 trial the opportunity to participate in the QoL sub-study.

### Analysis

Descriptive statistics using median and interquartile ranges for continuous variables and frequencies and percentages for binary and categorical variables were used to summarise the characteristics of the participants. The scores for each QoL measure were summarised for each timepoint using means and standard deviations or medians and interquartile ranges as appropriate. Distress Thermometer scores at baseline were categorised and compared across participant characteristics using a chi-squared test to identify factors that influenced the level of distress. To account for the repeated collection of QoL data from the same participants, longitudinal random effects regression models were used to assess whether QoL changed over time and differed across trial arms using all available data. Characteristics, including age, oestrogen receptor (ER) status, surgery type, disease type, hormone therapy use at randomisation, chemotherapy use and human epidermal growth factor receptor (HER2) status were also considered in a multivariate regression model to assess for independent predictors of high levels of distress.

A thematic content analysis [16] of the completed free text fields for all participants was carried out by SG using NVIVO version 12 software. Keywords that encapsulated the participant’s worries and concerns were identified from the free text and resulting themes developed. The themes were validated by a second independent researcher AB. Researcher were blinded to the trial arms whilst coding and only linked back to the trial arm data once content analysis was completed.

## Results

A total of 5235 women were randomised into the Mammo-50 trial between April 2014 and September 2018. 3858 (74%) participants were aged 60 years or older, 5084 (97%) were from a white ethnic background, 3279 (63%) had asymptomatic screen-detected tumour, 4202 (80%) had undergone conservation surgery, 4576 (87%) had invasive disease and 4330 (83%) had ER-positive tumours [11]. Of the 5235 women that were randomised, the majority (4751; 91%) consented to be in the QoL sub-study; 4488 (86%) women returned a baseline QoL booklet and a total of 4668 (89%) women returned at least one QoL booklet (Table 1). Given the majority of trial participants took part in the QoL sub-study, these were a representative sub-population of the main Mammo-50 trial [11].

**Table 1:** Form return

Timepoint	Annual Mammography *N* (%)	Less frequent mammography *N* (%)	Total randomised *N* (%)
Total randomised	2618	2617	5235
**Baseline (3 years post curative surgery)**	2260 (86.3)	2228 (85.1)	4488 (85.7)
**4 years**	2062 (78.8)	1886 (72.1)	3948 (75.4)
**5 years**	1924 (73.5)	1757 (67.1)	3681 (70.3)
**6 years**	1742 (66.5)	1594 (60.9)	3336 (63.7)
**7 years**	1670 (63.8)	1513 (57.8)	3183 (60.8)
**8 years**	1506 (57.5)	1418 (54.2)	2924 (55.8)
**9 years**	1103 (42.1)	993 (37.9)	2096 (40.0)
**10 years**	696 (26.6)	609 (23.3)	1305 (24.9)
**Number of patients with at least one form**	**2351 (89.8)**	**2317 (88.5)**	**4668 (89.2)**

*Note data collection for year 10 ongoing

**Fig. 1:**
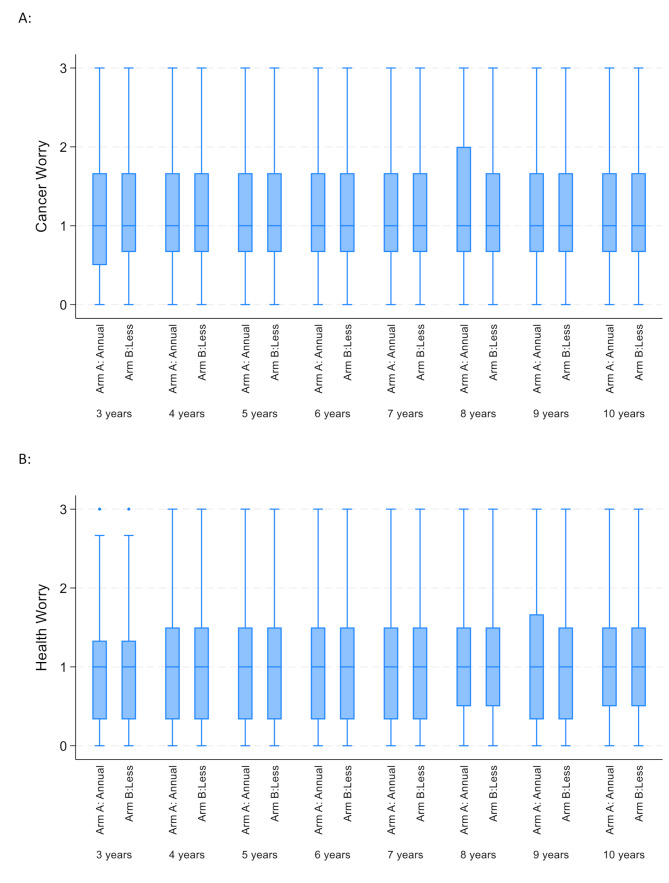
Assessment of survivor concerns (ASC) over time and by trial arm (Arm A: Annual mammography arm; arm B: less frequent mammography). **A**: Cancer Worry Scores; **B** Health Worry Scores

For all participants in the QoL sub-study, the median ASC cancer worry subscale score at baseline was 1 (Interquartile range 0.7–1.7) representing a “little bit” of concern (Table 2); no differences were seen between the trial arms (*p* = 0.60, Fig. 1A). Similarly, the median health worry subscale score at baseline was 1 (Interquartile range 0.3–1.3) representing a “little bit” of concern; no differences were seen between trial arms (*p* = 0.58, Fig. 1B). There was a small, yet statistically significant increase in cancer worry over time (β = 0.012; 95% CI 0.008–0.015, *p* < 0.001, Fig. 1A) and in health worry scores over time (β = 0.019; 95% CI 0.015–0.023, *p* < 0.0001, Fig. 1B).

**Table 2:** Summaries of quality-of-life scales at baseline

Characteristic	Annual Mammography	Less frequent mammography	Total randomised
**Total number with baseline form (%)**	2260 (86.3)	2228 (85.1)	4488 (85.7)
**Assessment of Survivor Concerns Fear of Recurrence**			
**Cancer worry subscale**			
Median (IQR)	1 (0.5–1.7)	1 (0.7–1.7)	1 (0.7–1.7)
Missing (n(%))	115 (5.1)	132 (5.9)	247 (5.5)
**Health worry subscale**			
Median (IQR)	1 (0.3–1.3)	1 (0.3–1.3)	1 (0.3–1.3)
Missing (n(%))	191 (8.5)	189 (8.5)	380 (8.5)
**The Warwick-Edinburgh Mental Well-being Scale (WEMWBS)**			
**WEMWBS score**			
Median (IQR)	54 (47–61)	54 (47–61)	54 (47–61)
Missing (n(%))	32 (1.4)	41 (1.8)	73 (1.6)
**Functional Assessment of Cancer Therapy - Breast**			
**Physical well-being subscale score**			
Median (IQR)	25 (22–27)	25 (22–27)	25 (22–27)
**Social/family well-being subscale score**			
Median (IQR)	25 (20–28)	25 (20–27)	25 (20–27)
**Emotional well-being subscale score**			
Median (IQR)	21 (18–23)	21 (18–23)	21 (18–23)
**Functional well-being subscale score**			
Median (IQR)	24 (19–26)	24 (20–26)	24 (20–26)
**Additional breast cancer concerns subscale score**			
Median (IQR)	30 (25–34)	30 (25–34)	30 (25–34)
**FACT-B Trial Outcome Index (TOI)**			
Median (IQR)	78 (68–85)	78 (68–85)	78 (68–85)
**FACT-G total score**			
Median (IQR)	93 (82–100)	92 (82–100)	92 (82–100)
**FACT-B total score**			
Median (IQR)	122 (108–133)	122 (108–132)	122 (108–132)
Missing (n(%))	72 (3.2)	71 (3.2)	143 (3.2)

**Fig. 2:**
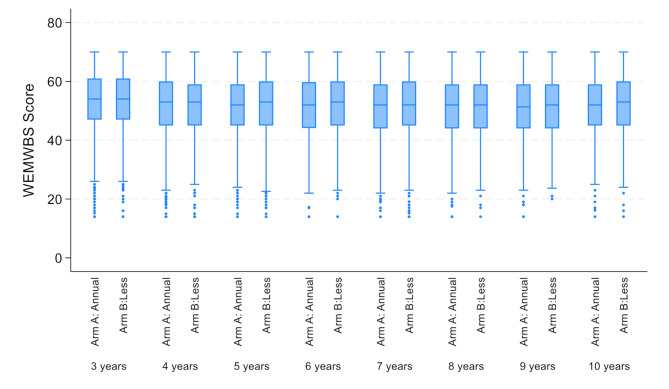
Warwick Edinburgh Mental Well-being Scale (WEMWBS) scores over time and by trial arm (Arm A: Annual mammography arm; arm B: less frequent mammography)

The median WEMWBS score at baseline was 54 (interquartile range 47–61) for both trial arms (Table 2). Patients’ mental well-being slightly worsened over time (β=-0.396; 95% CI: -0.442 to -0.351, *p* < 0.001), but did not differ across trial arms (β = 0.147; 95% CI: -0.377-0.670, *p* = 0.58, Fig. 2).

**Fig. 3:**
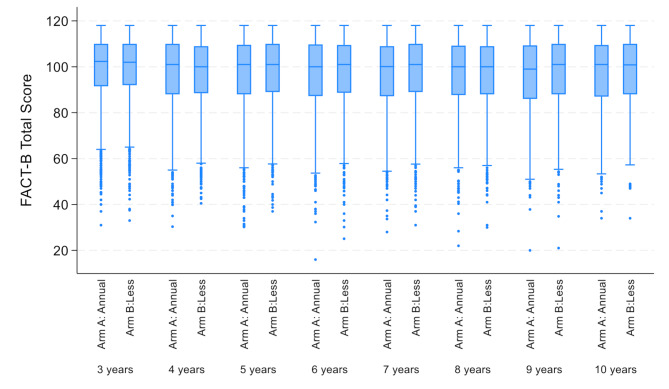
Functional Assessment of Cancer Therapy (FACT-B) Total Scores over time and by trial arm (Arm A: Annual mammography arm; arm B: less frequent mammography)

The median FACT-B total score at baseline was 122 (interquartile range 108–132); all subscales were similar across trial arms (Table 2). Participants’ QoL as measured by the FACT-B total score significantly decreased over time (β=-0.582; 95% CI: -0.644 to -0.520, *p* < 0.001). However, no statistically significant differences were found between trial arms (β = 0.294; 95% CI: -0.512-1.102, *p* = 0.47, Fig. 3).

**Fig. 4:**
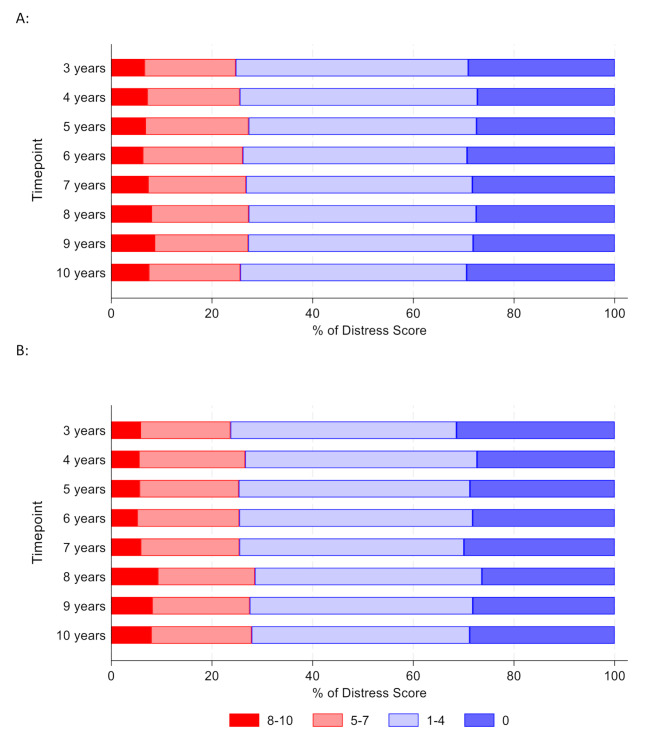
Percentage of distress scores over time split by trial arm; 1 A: for the Annual mammography arm; 1B: For the Less frequent mammography arm A:

Of the 4488 women returning a baseline QoL form, 3872 (86%) women completed the NCCN distress thermometer. A total of 1171 (30%) women had no levels of distress, 1764 (46%) had low levels of distress (score 1–4), 698 (18%) moderate levels of distress (score 5–7) and 239 women (6%) reported high levels of distress (score 8–10) at baseline (Table 3). There was a small, yet statistically significant, increase in distress scores over time (β_time_ = 0.05; 95% CI: 0.04–0.07, *p* < 0.001); however, the levels of distress did not significantly differ between the trial arms (β_arm_=-0.04; 95% CI: -0.16-0.08, *p* = 0.48, Fig. 4).

**Table 3:** Distress thermometer scores over time

Distress score	Baseline *N* (%)	4 years *N* (%)	5 years *N* (%)	6 years *N* (%)	7 years *N* (%)	8 years *N* (%)	9 years *N* (%)	10 years *N* (%)
**N randomised**	4488	3948	3681	3336	3183	2924	2096	1305
**N completed**	**3872**	**3354**	**3146**	**2851**	**2728**	**2532**	**1829**	**1136**
**0**	1171 (30.2)	916 (27.3)	883 (28.0)	821 (28.8)	793 (29.1)	683 (27.0)	515 (28.2)	331 (29.1)
**1–4**	1764 (45.6)	1565 (46.7)	1434 (45.6)	1295 (45.4)	1222 (44.8)	1143 (45.1)	814 (44.5)	502 (44.2)
**5–7**	698 (18.0)	660 (19.7)	634 (20.2)	572 (20.1)	533 (19.5)	489 (19.3)	347 (19.0)	216 (19.0)
**8–10**	239 (6.2)	213 (6.3)	195 (6.2)	163 (5.7)	180 (6.6)	217 (8.6)	153 (8.4)	87 (7.7)
**Missing**	616	594	535	485	455	392	267	169

The use of hormone therapy at randomisation was the only characteristic that significantly affected participants categorised levels of distress at baseline (*p* = 0.007, Table 4). Those participants that had stopped hormone therapy prior to enrolment onto the Mammo-50 study reported higher levels of distress (34% with moderate to high levels of distress) compared to those that were continuing to take hormone therapy (24%) and those that never took hormone therapy (24%; Table 4). The results of the multivariate modelling for predicting high levels of distress identified disease type (β = 0.400; 95% CI: 0.106–0.694, *p* = 0.008) and hormone therapy at randomisation (β = 0.124; 95% CI: 0.014–0.235, *p* = 0.027) as the only significant factors. Higher levels of distress were associated with invasive disease and never taking hormone therapy.

**Table 4:** Disease and treatment characteristics for the randomised patients by distress thermometer scores at baseline

Characteristics	No distress (0) *N* (%)	Low (1–4) *N* (%)	Medium (5–7) *N* (%)	High (8–10) *N* (%)	Chi-square *P*-value
**Total for randomised**	1171 (30.2)	1764 (45.6)	698 (18.0)	239 (6.2)	
**ER status**					0.281
ER negative	146 (34.5)	183 (43.3)	75 (17.7)	19 (4.5)	
ER positive	943 (29.4)	1475 (46.0)	580 (18.1)	206 (6.4)	
Not done	82 (33.5)	106 (43.3)	43 (17.6)	14 (5.7)	
**Surgery type**					0.256
WLE	927 (29.7)	1447 (46.3)	558 (17.9)	192 (6.2)	
Mastectomy	244 (32.6)	317 (42.4)	140 (18.7)	47 (6.3)	
**Disease type**					0.597
DCIS	158 (32.7)	209 (43.3)	89 (18.2)	28 (5.8)	
Invasive	1013 (29.9)	1555 (45.9)	610 (18.0)	211 (6.2)	
**Hormone therapy**					0.007
On-going	847 (30.1)	1304 (46.3)	487 (17.3)	178 (6.3)	
Stopped	44 (21.9)	89 (44.3)	52 (25.9)	16 (8.0)	
Never	280 (32.8)	371 (43.4)	159 (18.6)	45 (5.3)	
**HER2 status**					0.414
Positive	125 (31.8)	177 (45.0)	71 (18.1)	20 (5.1)	
Negative	879 (29.4)	1382 (46.3)	537 (18.0)	189 (6.3)	
Not done	167 (33.9)	205 (41.7)	90 (18.3)	30 (6.1)	
**Chemotherapy (CT)**					0.950
Yes	330 (30.1)	506 (46.2)	193 (17.6)	67 (6.1)	
No	838 (30.3)	1256 (45.3)	504 (18.2)	172 (6.2)	
Missing	3 (50.0)	2 (33.3)	1 (16.7)	0	
**CT containing taxane**					0.972
Without taxane	144 (29.9)	225 (46.7)	82 (17.0)	31 (6.4)	
With taxane	184 (30.1)	280 (45.8)	111 (18.2)	36 (5.9)	
Not specified	2 (66.7)	1 (33.3)	0	0	

The most common reasons from the problem list reported for causing high levels of distress (score of 8–10) at baseline, for the 239 participants, were fatigue, exhaustion or extreme tiredness (135 (56%) participants), sleep problems and/or nightmares (134 (56%) participants), worry, fear or anxiety (113 (47%) participants), hot flushes (96 (40%) participants), pain (84 (35%) participants), memory loss or concentration (83 (39%) participants), and sadness or depression (83 (35%) participants); these concerns remained constant over time (Table 5).

**Table 5:** The most common reasons for causing high levels of distress in all patients over time

Most common reasons for high level distress (8–10)	Baseline *N*(%)	4 years *N*(%)	5 years *N*(%)	6 years *N*(%)	7 years *N*(%)	8 years *N*(%)	9 years *N*(%)	10 years *N*(%)
**N**	239	213	195	163	180	217	153	87
**Fatigue & exhaustion**	135 (56.5)	128 (60.1)	103 (52.8)	95 (58.3)	91 (50.6)	112 (51.6)	80 (52.3)	45 (51.7)
**Sleep problems and/or nightmares**	134 (56.1)	117 (54.9)	89 (45.6)	72 (44.2)	75 (41.7)	106 (48.8)	69 (45.1)	42 (48.3)
**Worry**,** fear or anxiety**	113 (47.3)	122 (57.3)	107 (54.9)	95 (58.3)	101 (56.1)	124 (57.1)	80 (52.3)	47 (54.0)
**Hot flushes**	96 (40.2)	85 (39.9)	63 (32.3)	42 (25.8)	43 (23.9)	49 (22.6)	31 (20.3)	12 (13.8)
**Pain**	84 (35.1)	70 (32.9)	58 (29.7)	70 (42.9)	61 (33.9)	71 (32.7)	47 (30.7)	25 (28.7)
**Memory or concentration**	83 (34.7)	80 (37.6)	72 (36.9)	63 (38.7)	72 (40.0)	81 (37.3)	55 (35.9)	23 (26.4)
**Sadness or depression**	83 (34.7)	87 (40.8)	92 (47.2)	70 (42.9)	76 (42.2)	91 (41.9)	68 (44.4)	32 (36.8)

### Thematic content analysis

**Fig. 5:**
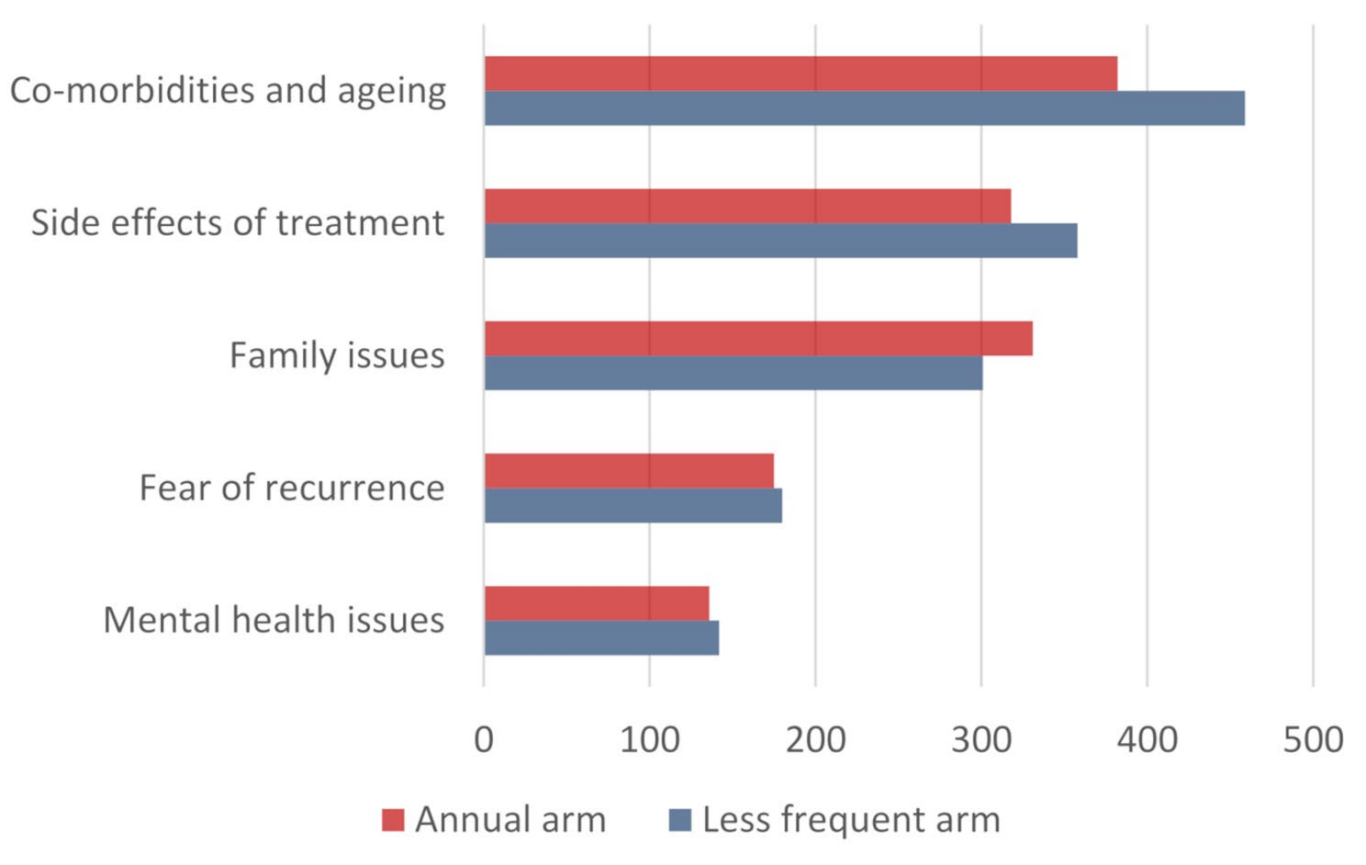
Frequency of overarching themes identified within the free text fields for the patient reported experiences

A total of 5125 free text fields were completed from 2581 participants (1304 participants on the annual arm; 1277 participants on the less frequent arm), with some participants contributing at several time points. The thematic content analysis of the free text responses from the questionnaire identified the main themes for participants on both arms of the study as co-morbidities and ageing, side effects of treatment, family issues, fear of recurrence and mental health issues (Fig. 5).

Many of the respondents described how they were living with co-morbidities which caused them more problems than their sometimes long-forgotten breast cancer diagnosis, e.g. cardiac issues (Table 6). Arthritis and osteoarthritis were cited by many respondents.

**Table 6:** Selected quotes relating to the patient reported experiences from the free text field on the quality of life booklets

Co-morbidities and ageing (22% of text fields)
• *As I am 72 it may be difficult to separate symptoms*,* like fatigue and lack of strength (physical & mental)*,* from possible post cancer symptoms. [Annual Arm 3 year]* • *I have lost about a stone in 6 months. My IBS is causing me some distress*,* but not the breast cancer. [ Annual Arm 4 year]* • *My main problem now is I have an inoperable leaking heart valve….this stops me doing a lot of the things I did. [Annual Arm 10 year]* • *I am a 70 year old lady living on her own. Any difficulties I have are more related to my age rather than having had breast cancer [Less frequent arm year 5].* • *I enjoy life rarely think about my former illness I get a bit of pain and stiffness but mostly related to age as I am 76 and lack a bit of energy for the same reason. [Less frequent arm year 8].*
**Side effects of treatment (18% of text fields)**
• *In the area of my operation after 3 years I still experience ‘pulling’ around the breast and pain in my ribs*,* this bothers me sometimes. [ Annual Arm 3 year]* • *Would accept it [hormone therapy] again as a precaution but feel that the treatment has been worse than the problem. [ Annual Arm 5 year]* • *Apart from the pain in the reconstruction area*,* I rarely think about breast cancer now. I wish I hadn’t had the reconstruction*,* as it feels false & is still uncomfortable*,* but it does not affect my life. [Less frequent arm year 5].* • *I stopped taking Letrozole after 3.5 years. it made sleeping difficult - (hot flushes)*,* I also got trigger fingers which made gardening*,* especially pruning*,* painful and awkward [Less frequent arm year 4].*
**Family Issues (16% of text fields)**
• *My husband died……during the night after many years of continuing weakness following strokes*,* we were married 62 years. I think maybe my answers to the questionnaire may be coloured by my response to this*,* rather than actual cancer worries [ Annual Arm 10 year]* • *…many of my feelings about emotional wellbeing relate to loss and bereavement… At the time this completely erased all my feelings about my own illness. I am still learning to cope with loss. [ Annual Arm 4 year]* • *My husband… passed away. This has obviously affected my well being.* [Less frequent arm year 10]. • *My only problems are all related to having a daughter who had to move in with me. Her problems just keep on growing - and it is all draining me - physically*,* mentally and financially!!!* [Less frequent arm year 3]
**Fear of recurrence (9% of text fields)**
• *With hindsight I would have preferred not to be in the trial as each year I have had a mammogram and had an all-clear letter I felt confident I was doing okay. This year of course I did not have that satisfaction. [Less frequent Arm 4 year]* • *I often think about a recurrence and reflect back on the treatment I received which saved my life and hope I don’t have to go through it again. [Annual Arm 3 year]* • *I have never felt that after removal of the cancer*,* it was an illness. I prefer to be positive and enjoy life. [Annual Arm 10 year]* • *I am anxious that I may get cancer again* [Less frequent arm year 4]
**Mental Health Issues (7% of text fields)**
• *The mental wellbeing of cancer patients is not being considered at all both during and after treatment. Life does change*,* cannot work out what is normal and what is down to side and after effects of cancer [Annual Arm 4 year]* • *The one thing that has affected me since my diagnosis is my lack of s self-confidence - this has really had a major impact on my life and the way I feel mentally……. as soon as all the treatment was over I felt very down and this has led to depression (whether this is due to the side effects of some of the medication I take I am unsure) but it is a constant battle to motivate myself to do anything or to socialise…. [ Annual Arm 3 year]* • *Most of my health concerns are not related to cancer. Arthritis and mental health play a large part* [Less frequent arm year 4]. • *My temper seems to be at a higher level these days. It worries me because relationships at home are suffering*,* and I nearly lost a close friend because of my volatile temper. [Less frequent Arm 8 year]*

**Fig. 6:**
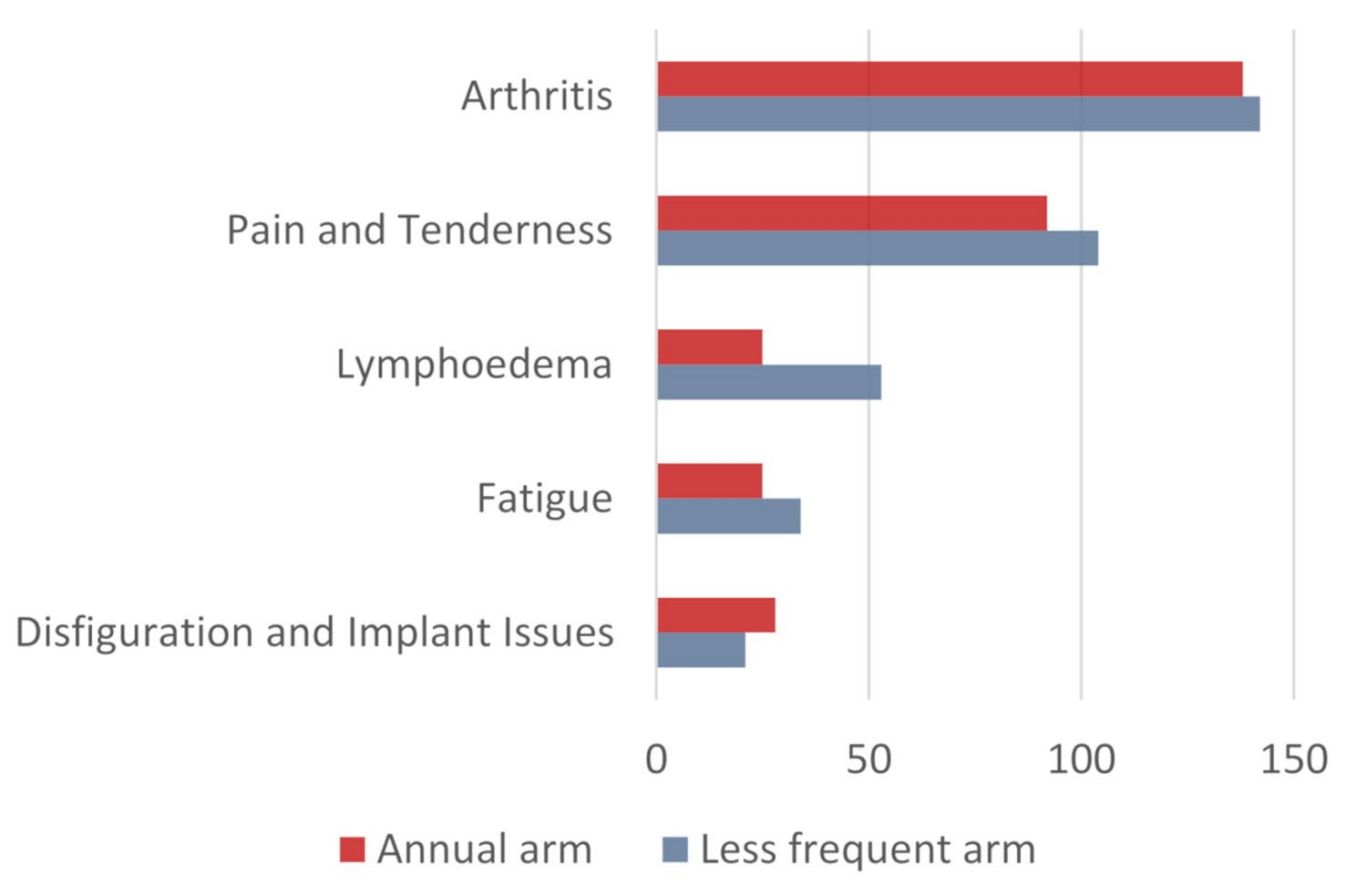
Frequency of side effects of treatment identified within the free text fields for the patient reported experiences

676 (18%) of the free text fields cited issues with side-effects of their cancer treatment (318 on the annual arm and 358 on the less frequent arm) (Fig. 5). The side effects most frequently described were arthritis, pain/tenderness, lymphoedema, fatigue and disfiguration/implant issues (Fig. 6; Table 6).

Family issues were cited in 632 (16%) free text fields (331 annual arm and 358 less frequent arm), (Fig. 5). Many of the reported family issues were inevitable consequence of living into older age, e.g. the death of a partner (Table 6).

355 (9%) free text fields described a fear of recurrence (175 annual arm and 180 less frequent) (Fig. 5). Participants in both arms of the study describe fear of recurrence and this fear is expressed at all timepoints of the study. Other participants stated that they really didn’t think about their breast cancer anymore.

Whilst 278 (7%) of the text fields cited issues with mental health (132 annual arm and 142 less frequent) (Fig. 5), reasons were often not directly related to the cancer diagnosis, but included family issues, bereavements and co-morbidities. Some of the participants report changes in their mood, often describing how difficult this can be to control. Finally, self-confidence is an issue for lots of participants (Table 6).

## Discussion

The Mammo-50 QoL sub-study explored QoL, fear of recurrence, distress levels as well as worries and concerns within a large randomised multicentre trial of annual versus less frequent mammographic surveillance. For this population of women aged 50 years or older at diagnosis of breast cancer and recurrence free at 3 years post diagnosis, there was no difference between trial arms for any of the QoL scales used in the study, suggesting that less frequent mammographic surveillance did not adversely impact QoL. The choice of validated QoL scales were informed in collaboration with the patient and public involvement group to reflect the issues such as fear of recurrence, mental health and well-being as well as functional assessment that were of importance to women with breast cancer in follow-up. In addition, the NCCN Distress Thermometer was used for patients to self-evaluate their levels of distress and its underlying causes [17, 18]. Within Mammo-50 the Distress Thermometer was a useful tool to identify participants with persistent unmet needs resulting from their diagnosis or their treatment for early breast cancer. Within our study, there was a small percentage of women who had high levels of distress (scores of 8–10) at baseline (6.2%), which was persistent over time (7.7% at 10 years), and the reasons associated with this distress was reported back to the clinical teams at site. In any de-escalation trial there needs to be some way of managing this small percentage of women longer term.

The differences found across time with increases in the distress scores, survivor concerns and cancer worry could be attributable to the very large numbers assessed in this study (i.e. the ability to detect very small differences) or it could reflect the patients’ worry increasing over time. It was reassuring that the median WEMWBS score of 54 in each trial arm was similar to the median score of 51 in the general population implying that for the majority of women they did not have impaired mental health or wellbeing. Existing research confirms that women with breast cancer experience higher levels distress, side effects and fear of recurrence, throughout their treatment and follow up [19]. Those with high levels of distress experience a poorer quality of life and survival rates [20]. Worry was the most frequently selected reason for the distress in our study. Fatigue and sleep issues were another physical problem experienced by women with breast cancer, causing increased distress [20, 21]. Osteoporosis is one of the most common side effects of cancer treatment which can greatly impact a patients QoL [22], along with long term physical changes such as chronic pain and anatomic changes [23].

This study is unique as it includes one of the largest collections of PROMs data [4, 5] and direct speech, reported in the free text, shared by breast cancer survivors, predominantly after conservation surgery and gathered over seven years, after the peak of clinical events. The voices in this report come mostly from participants benefiting from conservation surgery, with few making reference to the emotional morbidity of disfigurement. Both the Qol and the free text data support that women on both arms of the trial experienced similar levels of distress and worries. Participants irrespective of the frequency of mammographic surveillance indicated within the free text field that they were worried more about family issues and co-morbidities than their breast cancer. Fewer participants said they were worried about fear of recurrence than about other issues, regardless of which arm of the study they were on. Our study has highlighted that mental health is a constant struggle for many of these women as they cope with their personal history of breast cancer whilst supporting their loved ones as they move into older age.

Many of the side effects from the treatment this group of patients receive, especially taxanes and hormone treatment, can manifest in the same way as general effects of ageing and menopause: including fatigue, muscle aches and pains, increased body fat, hot flushes, sleepless nights [24]. Many of the women in the trial received aromatase inhibitors, prescribed to lower the natural levels of oestrogen after the menopause, but these are often responsible for arthritic side effects [24]. However, as this cohort of women could also be going through the menopause and naturally ageing, it is difficult to ascertain whether reported co-morbidities and side effects can be directly attributed to their hormone therapy or due to the ageing process.

To discontinue the hormone treatment means increasing the risk of recurrence and so women often prefer to continue and simply accept the side effects [25, 26]. The worry of stopping hormone therapy early may be reflected in the Mammo-50 participants reporting 10% higher levels of distress for those stopping treatment early compared to those who have never been on hormone therapy or those whose hormone therapy is on-going, albeit this group was quite small. Aging and menopause bring a whole range of inevitable symptoms, but it is unwise to dismiss them as not distressing [27]. Aging has a particular significance to cancer patients, it heralds a physiological decline in host immunity and immunosurveillance of occult disease [28]. A patient in self-care many years post diagnosis might best be prompted to take steps to prolong life by adhering to oestrogen deprivation measures and improving immunity by exercise, diet and avoiding smoking or alcohol [5, 29].

The Mammo-50 QoL sub-study provided a wealth of QoL data and patient reported experiences on the long-term survivorship of a large population of women with breast cancer. However, the trial was limited to those aged 50 years or older at diagnosis and at lower risk due to having to be recurrent free at three years post diagnosis and despite the trial being pragmatic majority of the participants recruited were from a white ethnic background and had small, lower grade ER positive tumours [11]. Thus as the results of this QoL substudy may not be generalisble to younger women diagnosed under 50, those at a higher risk, i.e. triple negative cancer, or those from other ethnic backgrounds that were not well represented within the Mammo-50 trial populations, further research is required.

## Conclusions

For women aged 50 years or older and 3-years post breast cancer diagnosis, less frequent mammograms (2-yearly after conservation surgery; 3-yearly after a mastectomy) compared to annual mammograms did not impact on their QoL or their reported experiences. For this group of ageing women, the Distress Thermometer provided a useful triaging tool that identified a small percentage of women with raised levels of distress even ten years after their diagnosis of breast cancer. These women may benefit from additional intervention whether this be psychosocial or management of ongoing hormone therapy side-effects or management of new co-morbidities. The results of this study add to the available evidence and help to inform clinical practice regarding the use of less frequent mammographic surveillance for this population.

## Data Availability

Data collected for the study, including individual participant data and a data dictionary defining each field in the set, will be made available to others subject to approvals. Request for access should be made to Trial team at Warwick Clinical Trials Unit. After approval, a signed data sharing agreement will be required prior to data release.

## References

[1] : Facts and Figures, Cancer B, May UK. 2024 [Available from: https://www.breastcanceruk.org.uk/about-breast-cancer/facts-figures-and-qas/facts-and-figures/

[2] Bray F, Laversanne M, Sung H, Ferlay J, Siegel RL, Soerjomataram I, Jemal A. Global cancer statistics 2022: GLOBOCAN estimates of incidence and mortality worldwide for 36 cancers in 185 countries. Cancer J Clin. 2024;74(3):229–63. 10.3322/caac.21834 38572751

[3] Mokhatri-Hesari P, Montazeri A. Health-related quality of life in breast cancer patients: review of reviews from 2008 to 2018. Health Qual Life Outcomes. 2020;18:1–25. 33046106 10.1186/s12955-020-01591-x PMC7552560

[4] Corner J, Wagland R, Glaser A, Richards SM. Qualitative analysis of patients’ feedback from a proms survey of cancer patients in England. BMJ Open. 2013;3(4):e002316. 23578681 10.1136/bmjopen-2012-002316 PMC3641435

[5] Marzban S, Shokravi S, Abaei S, Fattahi P, Karami M, Tajari F. Patient-Reported outcome measures of breast Cancer surgery: evidence review and tool adaptation. Cureus14(8):e27800. 10.7759/cureus.27800 PMC9481225 36134055

[6] Maheu C, Singh M, Tock WL, Eyrenci A, Galica J, Hébert M et al. Fear of Cancer recurrence, health anxiety, worry, and uncertainty: A scoping review about their conceptualization and measurement within breast Cancer survivorship research. Front Psychol. 2021;12. 10.3389/fpsyg.2021.644932 PMC8072115 33912113

[7] Götze H, Taubenheim S, Dietz A, Lordick F, Mehnert-Theuerkauf A. Fear of cancer recurrence across the survivorship trajectory: results from a survey of adult long-term cancer survivors. Psycho-oncology. 2019;28(10):2033–41. 31364222 10.1002/pon.5188

[8] Tran TXM, Jung S-Y, Lee E-G, Cho H, Kim NY, Shim S, et al. Fear of Cancer recurrence and its negative impact on Health-Related quality of life in Long-term breast Cancer survivors. Cancer Res Treat. 2022;54(4):1065–73. 34883553 10.4143/crt.2021.835 PMC9582487

[9] Evans A, Dunn J, Donnelly PK. Mammographic surveillance after breast cancer. Br J Radiol. 2024;97(1157):882–5. 38450420 10.1093/bjr/tqae043 PMC11075979

[10] Wilson BE, Wright K, Koven R, Booth CM. Surveillance imaging after curative-intent treatment for cancer: benefits, harms, and evidence. JCO. 2024;42(19):2245–9. 10.1200/JCO.23.02475 38805665

[11] Dunn JA, Donnelly P, Elbeltagi N, Marshall A, Hopkins A, Thompson AM, et al. Annual versus less frequent mammographic surveillance in people with breast cancer aged 50 years and older in the UK (Mammo-50): a multicentre, randomised, phase 3, non-inferiority trial. Lancet. 2025;405(10476):396–407. 39892911 10.1016/S0140-6736(24)02715-6

[12] Gotay CC, Pagano IS. Assessment of survivor concerns (ASC): A newly proposed brief questionnaire. Health Qual Life Outcomes. 2007;5(1):15. 17352831 10.1186/1477-7525-5-15 PMC1828718

[13] Tennant R, Hiller L, Fishwick R, Platt S, Joseph S, Weich S, et al. The Warwick-Edinburgh mental well-being scale (WEMWBS): development and UK validation. Health Qual Life Outcomes. 2007;5:1–13. 18042300 10.1186/1477-7525-5-63 PMC2222612

[14] Brady MJ, Cella DF, Mo F, Bonomi AE, Tulsky DS, Lloyd SR, et al. Reliability and validity of the functional assessment of Cancer Therapy-Breast quality-of-life instrument. JCO. 1997;15(3):974–86. 10.1200/JCO.1997.15.3.974 9060536

[15] NCCN. Distress Management (Version1.2008) 2007 [Available from: http://www.nccn.org

[16] Naeem M, Ozuem W, Howell K, Ranfagni S. A step-by-step process of thematic analysis to develop a conceptual model in qualitative research. Int J Qualitative Methods. 2023;22:16094069231205789.

[17] Ownby KK. Use of the distress thermometer in clinical practice. J Adv Pract Oncol. 2019;10(2):175–9. 31538028 PMC6750919

[18] Roth AJ, Kornblith AB, Batel-Copel L, Peabody E, Scher HI, Holland JC. Rapid screening for psychologic distress in men with prostate carcinoma: a pilot study. Cancer. 1998;82(10):1904–8. 9587123 10.1002/(sici)1097-0142(19980515)82:10<1904::aid-cncr13>3.0.co;2-x

[19] Sun H, Lv H, Zeng H, Niu L, Yan M. Distress thermometer in breast cancer: systematic review and meta-analysis. BMJ Supportive Palliat Care. 2022;12(3):245–52. 10.1136/bmjspcare-2021-002960 33975827

[20] McFarland DC, Shaffer KM, Tiersten A, Holland J. Prevalence of physical problems detected by the distress thermometer and problem list in patients with breast cancer. Psycho-oncology. 2018;27(5):1394–403. 29315955 10.1002/pon.4631 PMC5948165

[21] Koch L, Jansen L, Herrmann A, Stegmaier C, Holleczek B, Singer S, et al. Quality of life in long-term breast cancer survivors– a 10-year longitudinal population-based study. Acta Oncol. 2013;52(6):1119–28. 23514583 10.3109/0284186X.2013.774461

[22] Nardin S, Mora E, Varughese FM, D’Avanzo F, Vachanaram AR, Rossi V et al. Breast Cancer survivorship, quality of life, and late toxicities. Front Oncol. 2020;10. 10.3389/fonc.2020.00864 PMC7308500 32612947

[23] Lovelace DL, McDaniel LR, Golden D. Long-Term effects of breast Cancer surgery, treatment, and survivor care. J Midwifery Women’s Health. 2019;64(6):713–24. 31322834 10.1111/jmwh.13012

[24] Tenti S, Correale P, Cheleschi S, Fioravanti A, Pirtoli L. Aromatase inhibitors—induced musculoskeletal disorders: current knowledge on clinical and molecular aspects. Int J Mol Sci. 2020;21(16):5625. 32781535 10.3390/ijms21165625 PMC7460580

[25] Hershman DL, Shao T, Kushi LH, Buono D, Tsai WY, Fehrenbacher L, et al. Early discontinuation and non-adherence to adjuvant hormonal therapy are associated with increased mortality in women with breast cancer. Breast Cancer Res Treat. 2011;126:529–37. 20803066 10.1007/s10549-010-1132-4 PMC3462663

[26] McCowan C, Shearer J, Donnan PT, Dewar JA, Crilly M, Thompson AM, Fahey T. Cohort study examining Tamoxifen adherence and its relationship to mortality in women with breast cancer. Br J Cancer. 2008;99(11):1763–8. 18985046 10.1038/sj.bjc.6604758 PMC2600703

[27] Santoro N, Roeca C, Peters BA, Neal-Perry G. The menopause transition: signs, symptoms, and management options. J Clin Endocrinol Metabolism. 2021;106(1):1–15. 10.1210/clinem/dgaa764 33095879

[28] Hong H, Wang Q, Li J, Liu H, Meng X, Zhang H. Aging, cancer and immunity. J Cancer. 2019;10(13):3021. 31281479 10.7150/jca.30723 PMC6590045

[29] Cannioto RA, Attwood KM, Davis EW, Mendicino LA, Hutson A, Zirpoli GR, et al. Adherence to cancer prevention lifestyle recommendations before, during, and 2 years after treatment for high-risk breast cancer. JAMA Netw Open. 2023;6(5):e2311673–e. 37140922 10.1001/jamanetworkopen.2023.11673 PMC10160875

